# URINARY MARKERS OF OXIDATIVE STRESS CORRESPOND TO INFECTION AND AGING IN WILD CHIMPANZEES

**DOI:** 10.1093/geroni/igz038.3275

**Published:** 2019-11-08

**Authors:** Nicole A Thompson, Emily Otali, Zarin Machanda, Martin Muller, Richard Wrangham, Melissa Emery-Thompson

**Affiliations:** 1 University of New Mexico, Department of Anthropology, Albuquerque, New Mexico, United States; 2 Kibale Chimpanzee Project, Fort Portal, Uganda; 3 Tufts University, Department of Anthropology, Medford, Massachusetts, United States; 4 University of New Mexico, Department of Anthropology, Albuquerque, United States; 5 Harvard University, Department of Human Evolutionary Biology, Cambridge, United States

## Abstract

Oxidative stress (OS) plays a central role in aging and results from a variety of stressors, making it a powerful measure of health and a way to examine phylogenetic variation in life history. However, few urinary OS markers have been examined under field conditions, particularly in primates, and their utility to non-invasively monitor acute vs. chronic conditions is poorly understood. In this study, we examined variation in 5 urinary markers of oxidative damage and protection under 5 validation paradigms in 37 wild, adult chimpanzees living in the Kibale National Park, Uganda. We used 925 urine samples to conduct both cross-sectional and within-individual analyses of responses to acute infection and variation with age. Markers of damage (8-OHdG, F-isoprostanes, MDA-TBARS, and neopterin) and total antioxidant capacity were generally positively correlated with one another. Within individuals, all markers responded to at least one if not both types of acute infection. Markers of damage also varied with age, particularly in individuals near death. Unlike in human and rodent tissues, DNA damage in urine decreased with age, both across and within individuals near death, suggesting a potential decline in DNA repair and/or metabolic rate during senescence. Our results suggest that OS can be measured using field-collected urine and may be useful for both short- and long-term indicators of health. Our results further confirm that using multiple markers and longitudinal sampling within individuals is the most productive approach for studies that seek to determine the role of OS in health and lifespan in long-lived organisms.

